# Increased IL-1β in stratum corneum as a marker of inflammation among central centrifugal cicatricial alopecia patients with pruritus: An observational study

**DOI:** 10.1016/j.jdin.2023.09.005

**Published:** 2023-09-23

**Authors:** Aditi Gadre, Taylor Dyson, Jonathan Lai, Crystal Aguh

**Affiliations:** a The Johns Hopkins University School of Medicine, Baltimore, Maryland; b Georgetown University School of Medicine, Washington, District of Columbia; c Eastern Virginia Medical School, Richmond, Virginia

**Keywords:** alopecia, central centrifugal cicatricial alopecia, cytokine, inflammation, itch, pruritus

*To the Editor:* Central centrifugal cicatricial alopecia (CCCA) is an inflammatory condition of the vertex scalp predominantly affecting Black women. In comparison to cicatricial alopecias like frontal fibrosing alopecia and lichen planopilaris, CCCA often presents without overt signs of disease activity, such as erythema.^1^ Pruritus has anecdotally been suggested as a sign of disease activity in CCCA patients,^1^ but this has not been studied. Regular hair washing has been associated with decreased pruritus as well as a reduction in inflammatory markers, interleukin 1 alpha (IL-1α) and its receptor antagonist.^2^ Through analysis of cytokine expression patterns, our group sought to understand if pruritus is associated with scalp inflammation in CCCA and assess the impact of increased shampoo frequency, a known anti-inflammatory process, on pruritus and inflammation.

In our observational study, we implemented non-invasive tape stripping of the vertex scalp exposed by parting the hair to detect cytokine levels. Twenty-three Black women who met clinical or histologic criteria for diagnosis of CCCA and 26 Black, female controls were recruited. Patients were excluded if they had been treated with systemic or intralesional immunomodulators or antibiotics within 30 days or topical steroids within 7 days. Patients were stratified by frequency of pruritus as well as washing frequency (Table I). Frequent hair washing was defined as occurring at least biweekly and infrequent as occurring less than monthly. Standard protocols were followed for sample extraction, centrifugation, and concentration via Luminex bead-based immunoassays, with subsequent analysis of cytokines (Table II).

**Table I:** Characteristics of patient population

Characteristic	CCCA, *n* = 23, *n* (%)	Control, *n* = 26, *n* (%)	*P* value
Age, y, mean	46.34	44.62	.5559
Frequent washing, *n* (%)	14 (61)	12 (46)	.371
Clinical erythema, *n* (%)	3 (13)	0 (0)	.057
Scalp pruritus, *n* (%)	15 (65)	12 (46)	.181

	CCCA			Control		
	Frequent washing	Infrequent washing	Total	Frequent washing	Infrequent washing	Total
Frequent pruritus	11	4	15	4	8	12
Infrequent or absent pruritus	3	5	8	8	6	14
Total	14	9	23∗	12	14	26†

Table indicates the characteristics of patients enrolled in this study including associated signs and symptoms.

Significant (*P* < .05).

*CCCA,* Central centrifugal cicatricial alopecia.

∗ Chi-square test comparing hair washing frequency and scalp pruritus in patients with CCCA; *P* = .094.

† Chi-square test comparing hair washing frequency and scalp pruritus in control patients; *P* = .225.

**Table II:** Scalp cytokines assessed and associated results

Cytokine	Hair washing frequency						Pruritus					
	CCCA			Control			CCCA			Control		
	Frequent washing (SD)	Infrequent washing (SD)	*P* value	Frequent washing (SD)	Infrequent washing (SD)	*P* value	Frequent pruritus (SD)	Infrequent or absent pruritus (SD)	*P* value	Frequent pruritus (SD)	Infrequent or absent pruritus (SD)	*P* value
IL-1α	1852.69 ± 659.01	2564.02 ± 493.22	.3973	1143.46 ± 243.11	3191.9 ± 588.5	.005∗	2264.49 ± 443.06	1880.82 ± 385.18	.6192	2785.65 ± 723.94	1784.3 ± 349.93	.2309
IL-1RA	156.44 ± 61.79	331.46 ± 168.55	.3523	90.04 ± 29.35	233.43 ± 57.5	.0385∗	296.16 ± 75.81	91.32 ± 31.08	.0971	211.09 ± 61.15	129.67 ± 41.35	.2832
IL-1β	19.24 ± 7.51	7.51 ± 2.34	.1561	8.46 ± 3.60	8.99 ± 2.17	.8998	20.1 ± 6.93	4.44 ± .94	.0412∗	8.42 ± 1.98	9.03 ± 3.36	.8761
IL-8	102.38 ± 31.88	118.07 ± 54.24	.8068	54.16 ± 18.94	80.11 ± 33.36	.5064	136.15 ± 40.08	56.73 ± 22.36	.0988	91.61 ± 37.39	48.00 ± 17.74	.3078
IFN-α	23.41 ± 5.08	25.71 ± 3.59	.7155	22.48 ± 4.07	31.71 ± 5.95	.2136	24.94 ± 4.71	23.11 ± 4.20	.775	30.05 ± 7.20	25.23 ± 3.47	.5538

Table indicates which scalp cytokines were detected above threshold, comparing relevant groups and sub-groups. Assays for cytokines *IFN-γ, TNF-α, IL-2, IL-4, IL-6, IL-10, IL-12, IL-13, IL-17A* were conducted but were not detectable above threshold and consequently not included in analysis.

*IFN-α*, Interferon alpha; *IFN-γ*, interferon gamma; *IL*, interleukin; *IL-1α*, interleukin 1 alpha; *IL-1β*, interleukin 1 beta; *IL-1RA*, interleukin 1 receptor antagonist; *SD*, standard deviation; *TNF-α*, tumor necrosis factor alpha.

∗ Significant (*P* < .05).

While patients with CCCA reported more frequent scalp pruritus compared to controls, the difference was not statistically significant (*P* = .181). Additionally, shampooing frequency did not affect the frequency of scalp pruritus in patients with CCCA (*P* = .094) or control patients (*P* = .225). However, in patients with CCCA, increased scalp pruritus was associated with higher levels of interleukin 1 beta (IL-1β, *P* = .0412), whereas in controls, scalp pruritus was not associated with increases in any cytokines tested. As expected, based on prior literature data, shampooing frequency in control patients did result in lower levels of both IL-1 receptor antagonist (*P* = .0385) and IL-1α (*P* = .005), but this trend was not observed in CCCA patients (*P* = .3523 and .0902, respectively).

Our study suggests that in CCCA patients, pruritus is associated with active scalp inflammation, specifically IL-1β. IL-1α is expressed constitutively at high levels in epithelial cells.^3^ Exfoliation of superficial epidermal cells during shampooing is expected to decrease IL-1α levels in healthy scalp, but this result is likely less pronounced in CCCA due to associated epidermal and dermal changes from scarring. In contrast, IL-1β is inducible, requiring cleavage by a protease, caspase-1, which is activated by a nucleotide-binding domain-like receptor protein 3 inflammasome.^3^ Interestingly, IL-1β has been shown to increase expression of protease activated receptor-2,^4^ a critical component of the cowhage itch pathway, which also has been implicated in CCCA.^5^ This study is limited by its sample size but identifies a potential therapeutic target and affirms utility of tracking pruritus in CCCA patients.

## Conflicts of interest

None disclosed.
